# Infectious disease diagnostic competencies of medical students as a fundamental basis for physicians’ resilience in addressing future infectious disease challenges

**DOI:** 10.3205/id000106

**Published:** 2026-05-26

**Authors:** Claudia Brandt, Karsten Becker, Sandra Ciesek, Ulf Dittmer, Volkhard A. J. Kempf, Bettina Löffler, Frauke Mattner, Klaus Pfeffer, Jonathan Jantsch

**Affiliations:** 1 Permanent Working Group on Training of Medical and Postgraduate Students and Continuing Medical Education, German Society for Hygiene and Microbiology, Hanover, Germany; 2 Institute of Medical Microbiology and Hospital Hygiene, Frankfurt University Medicine, Frankfurt, Germany; 3 Friedrich Loeffler-Institute of Medical Microbiology, Greifswald University Medicine, Greifswald, Germany; 4 Society for Virology, Aschaffenburg, Germany; 5 Institute of Medical Virology, Frankfurt University Medicine, Frankfurt, Germany; 6 Institute of Virology, University Hospital Essen, Germany; 7 Institute of Medical Microbiology, University Hospital Jena, Germany; 8 Chair for Hygiene and für Hygiene und Environmental Medicine, Witten/Herdecke University, Witten, Germany; 9 Institute of Medical Microbiology and Hospital Hygiene, University Hospital Dusseldorf, Germany; 10 Institute of Medical Microbiology, Immunology and Hygiene, University Hospital Cologne, Germany

## Abstract

Teaching in the field of “Medical Microbiology, Virology, and Hygiene” focuses on the development of clinical competence in infection medicine, primarily achieved through time- and cost-intensive practical courses, which often compete with other educational offerings. These courses must address the defining feature of transmissibility of infectious agents, with far-reaching consequences for individual patients, their environments, society, animals, and the broader ecosystem (One-Health concept). Understanding the complexity of pathogen-associated risks requires comprehensive knowledge in epidemiology, prevention, diagnostics, and therapy of infectious diseases. Ensuring long-term resilience in the medical profession against the challenges of infectious diseases can only be achieved through fostering infection-related competencies in medical students. Qualified infection diagnostics are a core competence for independent, evidence-based medical practice. Proficiency in rapid and appropriate infection diagnostics is indispensable, as it forms the foundation for rational antimicrobial therapy. This is critical in preventing the misuse of antibiotics, which is the primary cause of the development of multidrug-resistant pathogens. Additionally, rapid infection diagnostics play a central role in controlling outbreaks and interrupting infection chains. In virology, microbiology, and hygiene, prompt medical intervention is likewise essential, as the interpretation of infection diagnostics depends heavily on factors like the timing of infection, clinical context, and pre-existing conditions or vaccinations. Correct sample collection, selection of appropriate tests, and the critical evaluation of microbiological and virological findings are essential medical competencies, which cannot be shifted to self-learning formats but can only be acquired through structured, practice-oriented education including hands-on-training. These competencies are central to infection diagnostics, prevention, and therapy.

## Abstract (de)

Die Lehre im Fach „Medizinische Mikrobiologie, Virologie und Hygiene“ zeichnet sich durch die Vermittlung von infektionsmedizinischen Handlungskompetenzen aus. Dies geschieht zumeist in zeit- und kostenintensiven praktischen Kursen, die im Wettbewerb mit anderen Lehrveranstaltungen stehen. Die Lehrveranstaltungen müssen dabei zwingend das Alleinstellungsmerkmal der Übertragbarkeit von Infektionserregern mit den weitreichenden Auswirkungen auf den individuellen Patienten, das Patientenumfeld, die Gesellschaft, Tiere und Umwelt berücksichtigen (One-Health-Konzept). Das Verständnis für die Komplexität dieser erregerassoziierten Risiken erfordert ein belastbares Wissen zur Epidemiologie, Prävention, Diagnostik und Therapie von Infektionserkrankungen. Eine auch zukünftig gesicherte ärztliche Resilienz gegenüber den infektionsmedizinischen Herausforderungen ist nur über infektionsmedizinische Handlungskompetenzen bei Studierenden der Humanmedizin zu erreichen. Hierbei stellt das Verständnis für eine qualifizierte Infektionsdiagnostik eine Kernkompetenz für jegliche eigenverantwortliche Ausübung wissenschaftlich-begründeter und evidenzbasierter ärztlicher Tätigkeit dar. Kenntnis und Einsatz adäquater und schneller Infektionsdiagnostik sind unabdingbar, weil nur sie als Grundlage der rationalen antimikrobiellen Therapie den missbräuchlichen Antibiotikaeinsatz verhindern, der die Hauptursache für die Entwicklung multiresistenter Krankheitserreger ist. Darüber hinaus spielt eine schnelle Infektionsdiagnostik bei der Eindämmung von Ausbrüchen und Unterbrechung von Infektionsketten eine zentrale Rolle. Ein rasches ärztliches Handeln ist im hochdynamischen Kompetenzfeld der Virologie, Mikrobiologie und Hygiene gleichermaßen gefordert, da Aussagekraft und Interpretation der Infektionsdiagnostik stark vom Infektionszeitpunkt, dem klinischen Kontext sowie von Impfungen oder Vorerkrankungen abhängen. Die korrekte Probenentnahme, die Auswahl des geeigneten Testverfahrens sowie die kritische Bewertung mikrobiologischer und virologischer Befunde sind essenzielle ärztliche Handlungskompetenzen, die keinesfalls im Selbststudium, sondern nur in einer strukturierten praxisorientierten Ausbildung mit praktischen Übungen erworben werden können und zentrale Elemente der Infektionsdiagnostik, -prävention und -therapie darstellen.

## Expert’s opinion

Degree programs across all disciplines are under constant pressure to appropriately prepare students for the exponential increase in knowledge. However, the resources allocated for this purpose, as well as the available time, are subject to inter-disciplinary competition due to the limited nature of these resources, accelerating the search for alternative teaching and self-learning formats. This can easily lead to the displacement of essential educational content, particularly resource- and time-intensive practical courses. Among these are laboratory-based subjects, particularly Medical Microbiology, Virology, and Hygiene, which involve pathogen-related and infection medicine content. The transmissibility of infectious agents, as a defining feature of infectious diseases, as well as the high dynamics of pathogens in terms of their spread, virulence, and resistance characteristics, necessitate both a patient-specific and a societal approach to the management of infectious diseases.

Given the challenges posed by an ever-changing world, driven in part by demographic processes, climate change, biodiversity loss, the convergence of human and animal habitats, migration, and threats from terrorism and warfare, infectious diseases can quickly have profound societal impacts, as the SARS-CoV-2 pandemic demonstrated. Recent years have seen pandemic and epidemic events heavily influenced by viral infections, including respiratory viruses, hemorrhagic fever viruses, and emerging zoonoses [1], [2], [3]. The ability to perform scientifically based infection diagnostics is therefore crucial not only for individual patient care but also as a foundation for outbreak detection, surveillance, contact tracing, and health policy decisions. Doctors must be trained to assess microbiological and virological findings not only for individual patients but also within the context of public health. This includes dealing with challenges arising from medical-technological advancements (e.g., the use of medical devices) and demographic shifts (e.g., an increase in young, elderly, multimorbid, and immunosuppressed patients), which can adversely affect the spread and progression of infectious diseases.

Another significant challenge is the so-called “silent pandemic” of antibiotic-resistant bacteria and other microorganisms, which are rapidly spreading worldwide. According to the Robert Koch Institute (RKI), an estimated 9,600 people in Germany died from infections with antibiotic-resistant pathogens in 2019, and 45,700 deaths were associated with such infections [4]. This increase in both community-acquired and nosocomial infections caused by multidrug-resistant bacterial pathogens represents one of the greatest global threats which the WHO already warned about urgently in 2014 using the term “post-antibiotic era” [5]. Parallel to this, the number of infections as primary diagnoses in intensive care units continues to rise [6], with over 20% of all hospitalized patients being treated for infections [7]. The rise in antibiotic resistance is considered one of the greatest global health threats, with estimates attributing 1.27 million deaths annually worldwide directly to multidrug-resistant pathogens and contributing to another 4.95 million deaths [8]. However, modern infectious medicine can only be an effective tool in fostering resilience to these threats if medical students are provided with a comprehensive understanding of the epidemiology, pathogenesis, diagnostics, therapy, and prevention of infectious diseases. Likewise, virology plays a special role, as it uniquely combines diagnostic, clinical, and public health perspectives and dynamics. It provides insight into the emergence of viral variants, mutation dynamics, and immune escape mechanisms, which are essential for the effectiveness of many vaccines. All of the above-mentioned content is indispensable for modern infectious medicine and must not be marginalized or outsourced to digital self-learning formats during potential reform processes.

A lack of competencies in pre-analytical, analytical, critical evaluation, and therapeutic implementation of infection diagnostic findings can lead to misdiagnoses and patient mistreatment. For example, improper diagnosis of urinary tract infections [9] and in particular of sepsis [10] can significantly increase the mortality rate of patients. Furthermore, future microbiological and virological point-of-care testing is increasingly being shifted to outpatient care, requiring supervision by qualified personnel and adequate quality management. The scientifically based interpretation of these point-of-care test results as well as microbiological and virological emergency diagnostics, cannot be solely provided by clinical microbiologists, and virologists but must also be carried out by general practitioners and other medical disciplines, particularly in rural areas. The practical competencies in essential infection diagnostic procedures and methods, which are crucial for effective patient care, cannot be acquired through self-study and must remain an integral part of the emergency diagnostic curriculum in medical school education.

As infectious diseases affect not only the infected patient but also their environment and can pose a vital threat to public health, students must be trained to approach infection-related medical actions with a holistic view, encompassing other patients, staff, domestic and wild animals, and the environment. They need to be aware in a One-Health context that pathogen transmissions can evolve from localized outbreaks into epidemic or even pandemic events. In terms of anti-infective therapy concepts, this means considering the avoidance of resistance development and resistance selection, as well as the significant socio-economic impacts of acute and chronic infections on health systems. Moreover, knowledge of the corresponding legal frameworks (e.g., reporting obligations, isolation requirements, hygiene regulations) is mandatory for all medical practice. Thus, only a solid infection diagnostic education during medical studies ensures that we are adequately prepared for the lethal threat posed by resistant pathogens, foodborne pathogens (e.g., EHEC), pandemic viruses (e.g., SARS-CoV-2), and highly resistant fungi (e.g.,* Candida auris*).

Newer educational concepts define an increasingly demanding and comprehensive graduate profile for medical students, aiming to ensure the employability and competence of graduates in various medical roles after a six-year medical curriculum. These concepts require that young doctors increasingly base their daily work on scientific findings and apply the best available evidence according to evidence-based medicine principles. In the ongoing discussion about medical education reform in Germany, there is a tendency to allow considerable flexibility to medical faculties in designing curricula. This could lead to substantial portions of content being shifted to self-study or digital learning formats, resulting in insufficient acquisition of infection-related knowledge and skills. However, these are indispensable in infection medicine for fostering critical and analytical thinking, ensuring that students can responsibly perform medical tasks in any discipline, independent of their specialization. The elimination of practical skills related to microorganisms (bacteria, viruses, fungi, parasites) would be particularly detrimental, since for scientifically based treatment of infectious diseases, it is necessary to provide specific microbiological or virological evidence, which alone forms the basis for targeted infectious disease treatment [11].

The educational content in Medical Microbiology, Virology, and Hygiene focuses on pathogens, their characteristics, epidemiology, and interactions with their hosts, thereby shaping the understanding of the specific manifestations of diseases caused by infectious agents. This knowledge is not only crucial in infectious differential diagnostics but also for selecting appropriate diagnostic materials, laboratory confirmation of infections, and for the therapy and prevention of infectious diseases. Mastering of core contents in infection diagnostics (e.g., epidemiology, pathogenesis, resistance mechanisms, diagnostic stewardship), infection prevention (e.g., hygiene, vaccinations), and infection therapy (e.g., antibiotics, antiviral therapies, immunotherapies, antibiotic stewardship) has been legally mandated in Germany since 2011 through the Infection Protection Act (§23) [12]. These competencies can reliably be conveyed only through independent, practical infection diagnostic exercises using relevant case examples in medical school education.

Only under direct supervision and guidance from medically specialized staff students can learn the central principles and specificities of diagnostics, as well as the subsequent therapeutic and hygienic consequences, through practical exercises. Simultaneously, they will be taught safe, responsible handling of infectious patient materials and potentially pathogenic agents, as well as the responsible use of antibiotics. Moreover, the personal understanding of increasingly complex diagnostic decision-making processes will be promoted, preparing students to transfer developed solutions to similar situations and challenges. A deep understanding of the importance and scope of these theoretical and practical skills is essential for ensuring that future medical professionals will choose to work in this fundamental area of medicine [13].

In all reform processes of medical education, it is crucial to ensure that the content of Medical Microbiology, Virology, and Hygiene continues to be taught in the practical context of specialists in microbiology, virology, infection epidemiology, and hygiene, as part of compulsory practical courses. Only in this way it can be ensured that graduates will continue to possess the secure and sound competencies necessary to protect not only individual patients but also to safeguard public health and well-being.

## Notes

### Use of AI

During the preparation of this work, the authors used ChatGPT AI (https://chat.openai.com/) in order to improve English language and grammar. After using this tool/service, the authors reviewed and edited the content and take full responsibility for the content of the publication.

### Competing interests

The authors declare that they have no competing interests.
